# Use of clomiphene citrate alone, urinary follicle-stimulating hormone alone, or both combined sequentially in patients with unexplained subfertility undergoing intrauterine insemination: A randomized trial

**DOI:** 10.4274/tjod.99835

**Published:** 2019-01-09

**Authors:** Reyhan Ayaz, Mehmet Reşit Aşoglu, Selçuk Ayas

**Affiliations:** 1 University of Health Sciences, Van Traning and Research Hospital, Clinic of Perinatology, Van, Turkey; 2 University of Maryland Medical Center, Clinic of Obstetrics and Gynecology and Reproductive Sciences, Baltimore, Maryland, USA; 3 Okan University Faculty of Medicine, Department of Obstetrics and Gynecology, İstanbul, Turkey

**Keywords:** Clomiphene citrate, urinary follicle-stimulating hormone, intrauterine inseminaton, unexplained subfertility

## Abstract

**Objective:**

To compare the successes of clomiphene citrate (CC) alone, pure human urinary follicle-stimulating hormone (uFSH) alone, and both combined sequentially in patients with unexplained subfertility couples undergoing intrauterine insemination (IUI).

**Materials and Methods:**

Patients aged 18-38 years who had a normal uterine cavity, at least one normal fallopian tube, and regular menses and were unable to conceive despite unprotected intercourse for at least 12 months were randomized to receive CC alone, uFSH alone, or sequential CC and uFSH before a single IUI. The primary outcomes were clinical pregnancy and live birth rates. The study was approved by the ethics committee of our institution.

**Results:**

A total of 135 patients were randomized, and 121 of these were able to complete the study. Of these, 30% (n=36) had CC alone, 34% (n=41) had uFSH alone, and 36% (n=44) had sequential CC and uFSH. The three groups did not significantly differ in terms of age, duration of infertility, hormone levels, and semen parameters. For CC alone, uFSH alone, and sequential CC plus uFSH groups, pregnancy rates were 8.3%, 17.1%, and 18.2%, respectively (p>0.05), and live birth rates were 8.3%, 12.1%, and 13.6%, respectively (p>0.05).

**Conclusion:**

In women with unexplained infertility, use of uFSH seemed to increase the success rate compared with CC alone. The sequential regime can significantly reduce the treatment cost if gonadotropin/IUI cycles are planned.

**PRECIS:** Administration of clomiphene citrate for 5 days appears reasonable in reducing the treatment cost for couples with unexplained subfertility who undergo gonadotropin/intrauterine insemination cycles.

## Introduction

Intrauterine insemination (IUI) following ovarian stimulation (OS) is a reasonable treatment modality in cases of unexplained subfertility, which is responsible for approximately 10-30% of infertility causes^(1,2,3)^. This approach will possibly maintain its place in the management of unexplained subfertility in the near future unless the cost of assisted reproductive technology (ART) is reduced to an acceptable level for most couples. Ideal OS treatment before IUI procedures is expected to minimize the likelihood of ovarian hyperstimulation and multiple pregnancies while increasing chance of live birth (LB) rates^(4,5)^. Although various empiric OS regimes exist to improve a couple’s chance of conceiving, a great deal of debate regarding the superiority of one regime over another has continued over the years^(6)^. Therefore, randomized studies are needed to address ongoing controversy and guide use in regard with the choice of treatment regimen. Clomiphene citrate (CC) and gonadotropin are well-established agents to induce follicular development. CC is commonly used as the initial agent for OS as it has certain advantages such as being affordable, patient friendly and well-tolerated, and does not require frequent monitoring. Women who remain anovulatory on CC or who have not conceived after several cycles are considered appropriate candidates for OS with gonadotropins. However, gonadotropin has higher cost compared with CC and requires multiple injections^(7)^. These disadvantages have raised a quest for alternative treatment protocols. For this purpose, a sequential CC/gonadotropin regimen has been considered as a potential alternative to reduce both treatment cost and gonadotropin dose used if IUI is planned^(8)^. We conducted this randomized trial to compare CC alone, pure human urinary follicle-stimulating hormone (uFSH) alone or both combined sequentially for OS followed by IUI in patients with unexplained subfertility of at least 12 months.

## Materials and Methods

This randomized trial included couples with unexplained subfertility who presented to the reproductive endocrinology and infertility clinic at Zeynep Kamil Training and Research Hospital, from January 2009 to March 2011. The ethics committee at our institution approved this study (approval number: 18145/07.12.2010). Each participant provided informed consent.

### Study population

For this study, diagnosis of unexplained subfertility was made after a standard infertility examination (including semen analysis, hysterosalpingogram and ovulation test) resulted in normal findings in couples who could not conceive despite unprotected intercourse for at least 12 months. On this basis, all male partners had a normal semen analysis and all female partners had at least one healthy fallopian tube, normal uterine cavity, and regular menses. To minimize heterogeneity and confounding factors in the study population, the following criteria were considered as the exclusion reasons: age of less than 18 or more than 38 years, secondary infertility, body mass index (BMI) above 30 kg/m^2^ or below 19 kg/m^2^, basal (day 3) FSH levels ≥14 IU/L, irreregular menses, any endocrine diseases, or history of use of gonadotropin, oral contraceptives or other hormonal drugs during the last 6 months.

### Patient evaluation

All participants were evaluated using a standard approach. On the second or third day of the menstrual period, transvaginal ultrasound (TVU) using a 6.5-MHz probe (Logic alfa 200 GE Medical A/S Milwaukee, United States) was performed to evaluate uterus size, endometrial thickness, adnexa and ovaries as well as count the number of antral follicles. The following hormonal and biochemical tests were obtained on the same day; estradiol (E_2_), FSH, luteinizing hormone (LH), prolactin, thyroid function tests, fasting blood sugar, and renal and liver function tests. Hysterosalpingography (HSG) was performed for the assessment of tubal patency after menstruation according to the literature-based recommendations, followed by laparoscopy if HSG was inconclusive. The standard evaluation also included age, BMI, duration of infertility, blood pressure, pelvic examination, cervical smear, vaginal-cervical cultures, and infection screening if needed. For the evaluation of male fertility, our andrology laboratory evaluated all semen samples using the World Health Organization and Kruger criteria^(9,10)^. The following parameters of semen analysis were accepted as the inclusion criteria: a concentration more than 15 million/mL, type A plus B motility higher than 40% and normal morphology more than 14%. Sperm preparation was performed soon after ejaculation based on swim up technique using Earle’s Balanced Salt (Sigma) and *in vitro *fertilization-30 (Vitrolife) (Merck Company) solutions.

### Study groups

An OS protocol coupled with IUI was the treatment of choice offered to couples diagnosed with unexplained infertility following the required gynecologic and laboratory evaluations. The study population was stratified into three groups; CC alone, uFSH alone, and combination of CC and uFSH (CC-uFSH). The first, second, and third study participant was included in the CC alone, uFSH alone, and sequential CC-uFSH groups, respectively, and this allocation sequence was followed until each group contained 45 patients (a total of 135 women).

### Treatment protocols

All the patients had a TVU assessment on the second or third day of the cycle. A CC dosage of 100 mg/day orally was initiated in the CC alone and sequential CC-uFSH groups, and an uFSH dose of 75 IU subcutaneously in the uFSH alone group. After a treatment course of five consecutive days, a follow-up TVU was performed in all patients. Afterwards, the CC alone group received no additional treatments, the sequential CC-uFSH group started administering daily uFSH injections with a dose of 75 IU as a sequential treatment, and the uFSH alone group continued the treatment protocol already being used. TVU monitoring was performed in all patients to check follicle growth at certain intervals until the day of human chorionic gonadotropin (hCG) injection, when the lead follicle reached ≥18 mm in the CC alone group and ≥17 mm in the uFSH alone and sequential CC-uFSH groups, which was followed by a single IUI procedure at around 36-40 hours after subcutaneous hCG injection (10.000 IU). The patients rested for 10 min following the IUI. No medications were used in the luteal phase. Criteria for cycle cancellation were as follows; spontaneous ovulation before the day of hCG, inadequate response to CC, and excessive response to uFSH or CC.

### Outcomes

The primary outcomes were clinically detectable gestation (CDG) and LB rates. CDG was defined as an intrauterine pregnancy with a positive heart beat detected by TVU at approximately six weeks of gestation. LB was defined as delivery of a living infant after 24 completed weeks of gestation. The secondary outcomes were miscarriage rates, endometrial thickness, number of dominant follicles, duration of treatment, and number of uFSH injections used. Miscarriage was defined as loss of a clinically detectable pregnancy before 20 weeks of gestation. Endometrial thickness (distance between both endometrial-myometrial junctions in sagittal plane 1 cm below the uterine fundus) measured on the day of hCG was used in the comparisons. These outcomes were compared among the three groups.

### Statistical Analysis

Data were analyzed using NCSS (Number Cruncher Statistical System) 2007 & Power Analysis and Sample Size 2008 (Utah, USA). One-way analyses of variance (ANOVA), Pearson’s chi-square, and the Student’s t-test were the statistical tests used for the comparisons. A p value of 0.05 was accepted as the cut-off for statistical significant. All values are given as mean ± standard deviation or percentage.

## Results

**Figure 1:**
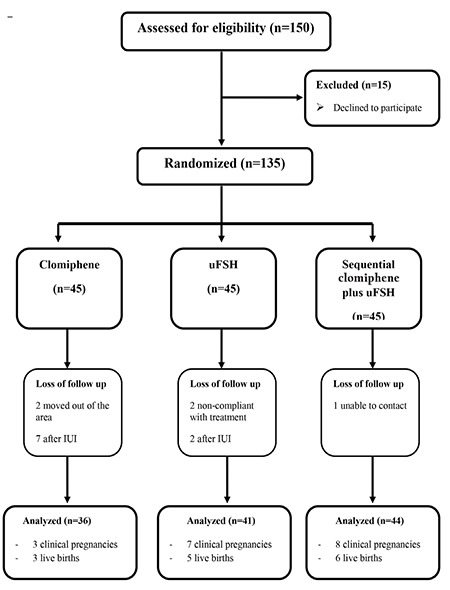
Flowchart of the study uFSH: Urinary follicle-stimulating hormone, IUI: Intrauterine insemination

A total of 150 women met the inclusion criteria of the study; 15 women declined to participate in the study. The remaining 135 women were randomized to a treatment group according to the sequence of clinical appointment, and 121 of those were able to complete the study. Of the 121 patients, 30% (n=36) had CC alone, 34% (n=41) had uFSH alone, and 36% (n=44) had sequential CC-uFSH (Figure 1). The rate of treatment cancellation was 2.2%, 6.6%, and 4.4% in the CC alone, uFSH alone, sequential CC-uFSH groups, respectively (p>0.05). The three groups did not significantly differ in terms of age, duration of infertility and basal FSH, LH, and E_2_ (Table 1). The groups also showed no statistical difference in the semen parameters (p>0.05, Table 2). Table 3 displays the outcomes of the treatment protocols. The duration of treatment was shorter in the CC alone group than in the uFSH alone or sequential CC-uFSH group (p<0.01), but there was no difference between the uFSH alone and sequential CC-uFSH groups. The consumption of uFSH was less in patients who took sequential CC-uFSH than in those who took uFSH alone (p<0.01). The number of mature follicle differed between the groups (p<0.01). The sequential CC-uFSH group had the highest number of mature follicle on the day of hCG. Endometrial thickness did not differ between the three treatment regimens (p>0.05). For the CC alone, uFSH alone, and sequential CC-uFSH groups, CDG were 8.3%, 17.1%, and 18.2%, respectively. LB rates were 8.3%, 12.1%, and 13.6%, respectively. The CC alone group had lower CDG and LB rates than the other groups, but this did not show statistical difference (p>0.05, Table 3). The rates of CDG and LB were quite similar between the uFSH alone and sequential CC-uFSH groups (Table 3). No miscarriage occurred in the CC group. On the other hand, miscarriage occurred in two of seven patients in the uFSH alone group and two of eight patients in the sequential CC-uFSH group; the miscarriage rates did not differ between these groups (p>0.05).

## Discussion

This randomized study provides the outcomes of couples with unexplained subfertility who underwent one session of IUI following OS with CC alone, uFSH alone, or sequential CC-uFSH. Based on our results, use of uFSH alone or sequential CC-uFSH as an OS protocol seemed to offer higher success rates compared with CC alone, although the difference was not significant. The sequential protocol had similar CDG and LB rates to uFSH alone. Although the sequential regimen increased the success rate compared with CC alone regimen, it reduced the number of uFSH injections used for OS compared with uFSH alone. Thus, the treatment cost could be estimated to be lower in the sequential regimen than in the uFSH alone regimen, without affecting the duration of treatment and success rate.

Although it is inconclusive as to whether ongoing pregnancy rates are higher with IUI (with or without OS) than in timely sexual intercourse according to the most recent Cochrane analysis^(11)^, clinical practice usually agrees on the use of oral agents or gonadotropin in IUI cycles to presumably improve the chance of conceiving in the presence of unexplained subfertility^(12)^. In a randomized study of 93 unexplained infertile patients who had a trial of OS/IUI, Berker et al.^(12)^ found that the rate of ongoing pregnancy was 11.6% (5/43) and 18% (9/50) when treatment regimen was CC and FSH, respectively. Dankert et al.^(13)^ showed that LB rates were respectively 31.4% and 30.3% in the CC and FSH groups among a total of 138 patients (68 couples with unexplained infertility, 70 couples with male subfertility) who underwent IUI up to four cycles. These studies did not reach statistical significance in the aforementioned outcomes, as with our study. In a comparison of two drugs, Diamond et al.^(14)^ reported that CC and gonadotropin groups had LB rates of 32.2% and 23.3% in a high number of patients with unexplained infertility who underwent OS/IUI for up to four cycles, respectively, and this difference achieved statistical significance. The patient populations and treatment protocols used in these studies were quite similar to our patient population. However, we think that a similar success rate was also able to be achieved with a sequential CC-uFSH regimen based on our results. Therefore, it may be reasonable to use CC priming to reduce the treatment cost before administrating gonadotropin. In a retrospective analysis of 648 IUI cycles, Ryan et al.^(15)^ revealed less cost and multiple pregnancies in a sequential oral medication (CC or letrozole or tamoxifen) and human menopausal gonadotropin (hMG) group than in an hMG alone group, with similar success rates in both groups. A recent metaanalysis including 22 studies showed use of CC to reduce FSH consumption during OS, with no change in clinical and LB rates [RR (relative risk), 1.0, (95% CI (confidence interval), 0.8-1.4) and RR, 0.9 (95% CI, 0.6-1.2) respectively]^(16)^. Endometrial thickness and pattern are considered to affect the success of IUI^(17)^. CC may cause adverse effects on endometrial quality due to its anti-estrogenic effects. Despite no statistical difference, the mean endometrial thickness was greater in the uFSH alone and sequential CC-uFSH groups than in the CC alone group in our study. This may show a benefit of using gonadotropin following CC because the success rates were similar in both the uFSH alone and sequential CC-uFSH groups, but lower in the CC alone group than in the others. Therefore, a sequential protocol may offer an advantage by correcting the possible adverse effects of CC on the endometrium.

There are some limitations in this study. First, the sample size was not large enough to draw definitive conclusions. Second, because the study did not have an expectant management group as a control group, the contribution of treatment protocols to clinical pregnancy or LB rates remained unclear. Last, the mean duration of infertility was relatively long because couples with unexplained infertility should undergo IUI procedure for at least two trials to be eligible for the use of ART in Turkey if they have insurance funded by the government to cover treatment expenses. The main strength of this study was its randomized design.

## Conclusion

In women with unexplained infertility, although clinical pregnancy and LB rates for IUI with CC, uFSH or sequential CC-uFSH did not reach statistical significance, use of uFSH seemed to increase the success rate compared with use of CC alone. Use of CC for 5-days before gonadotropin can significantly reduce the treatment cost if gonadotropin/IUI cycles are planned.

## Figures and Tables

**Table 1:**
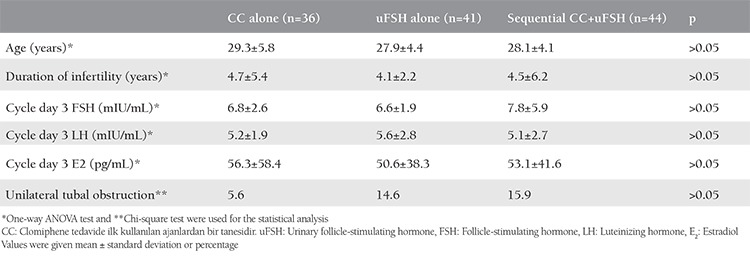
Comparison of patient characteristics between the groups

**Table 2:**
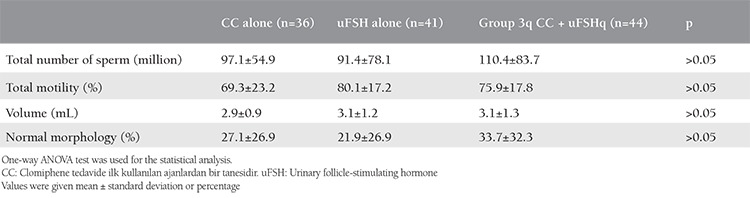
Comparison of sperm parameters between the groups

**Table 3:**
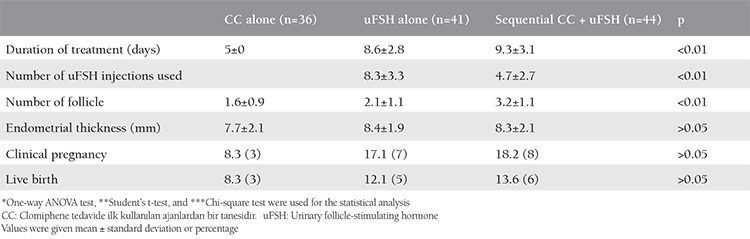
Comparison of the outcomes between the groups
